# Patterns of Infectious Disease Identified in Clinical Autopsy at a South African Tertiary Care Setting: A 10-Year Retrospective Study

**DOI:** 10.3390/diseases14060221

**Published:** 2026-06-19

**Authors:** Moshawa Calvin Khaba, Morongwa Dikotope, Thato Nkwagatse, Ramokone Maphoto, Thandekile Manzini, Khomotso Maaga, Ndivhuho Agnes Makhado

**Affiliations:** 1 Department of Anatomical Pathology, Sefako Makgatho Health Sciences University, Pretoria 0204, South Africa; morongwadikotope@gmail.com (M.D.); thato1.nkwagatse@nhls.ac.za (T.N.); 2 Dr George Mukhari Tertiary Laboratory, National Health Laboratory Service, Pretoria 0204, South Africa; ramokone.maphoto@nhls.ac.za; 3 Department of Virology, Sefako Makgatho Health Sciences University, Pretoria 0204, South Africa; 4 Infectious Disease Unit, Department of Internal Medicine, Dr George Mukhari Academic Hospital, Sefako Makgatho Health Sciences University, Pretoria 0204, South Africa; thandekileman@gmail.com; 5 Department of Public Health, Sefako Makgatho Health Sciences University, Pretoria 0204, South Africa; khomotso.maaga@smu.ac.za; 6 Department of Medical Microbiology, Sefako Makgatho Health Sciences University, Pretoria 0204, South Africa; nmakhado@yahoo.com

**Keywords:** prevalence, autopsy, infections, HIV/AIDS

## Abstract

Background: Infectious diseases remain a leading cause of mortality in South Africa, compounded by a high HIV prevalence. This study aimed to delineate the spectrum and clinicopathological characteristics of fatal infectious diseases through a postmortem audit to inform clinical practice and public health strategy. Methods: A retrospective, cross-sectional descriptive study was conducted on all autopsies with a final cause of death attributed to infectious disease at a National Health Laboratory Service, in Northern Pretoria, Gauteng, South Africa, from 2012 to 2021. Using the Systematised Nomenclature of Medicine Clinical Terms (SNOMED) code and word search engines codes, 55 cases were identified. Data on demographics, clinical presentation, HIV status, antiretroviral therapy (ART), comorbidities, and final autopsy diagnosis were extracted from the laboratory information system. Histological confirmation was performed using standard stains. Descriptive statistical analysis was conducted using STATA-18. Results: The cohort (n = 55) had a median age of 31 years (IQR 19–45) and was predominantly female (67%). HIV prevalence was 35%, with 68% of those on ART. The leading cause of death was multilobar pneumonia (36%), followed by bronchopneumonia (22%). AIDS-defining illnesses were present in 27% of cases, with disseminated tuberculosis being the most common (46%). Septic shock was identified in 18% of decedents. A significant proportion (60%) of the cohort was HIV-negative. Conclusions: This autopsy series reveals a high burden of fatal community-acquired pneumonias and HIV-associated opportunistic infections, with a notable proportion of deaths occurring in HIV-negative individuals. The findings underscore diagnostic gaps and highlight the critical role of autopsy in accurate mortality surveillance, advocating for enhanced antemortem diagnostic protocols and targeted public health interventions.

## 1. Introduction

South Africa continues to bear a substantial burden of infectious diseases, which persist as leading causes of mortality. The fact that many of these conditions are both preventable and treatable highlights persistent gaps in care and prevention strategies, pointing to opportunities for strengthening public health systems [1].

Infectious diseases are caused by pathogenic microorganisms, including bacteria, viruses, fungi, protozoa, helminths, and prions [2]. While they can affect all individuals, their prevalence and severity are profoundly exacerbated by determinants of health, such as low socioeconomic status, overcrowding, poverty, and rapid urbanisation, which are highly prevalent in many South African communities [2,3]. Globally, the annual incidence of infectious diseases has risen since 2001, with the African region reporting the highest numbers [4]. South Africa’s burden is compounded by the world’s largest HIV/AIDS epidemic, which significantly increases susceptibility to a wide range of opportunistic and non-opportunistic infections [5]. However, the introduction of antiretroviral therapy (ART) has transformed HIV from a fatal disease into a manageable chronic disease with a significant reduction in AIDS-related illness, but inequities in access, late presentation and treatment failure still result in preventable mortality in South Africa [6].

A great challenge in managing this load is the varied, often unexpected way infections present. Many are without symptoms or come with general clinical features, resulting in delayed or missed diagnoses, with the cause of death only being discovered after death during autopsy [7,8,9]. In this situation, the autopsy still remains the best tool for finding out the cause of death, even though there has been a worldwide decrease in the rates of autopsies [7]. The underutilisation of autopsies represents a significant public health concern, as it results in the uncounted burial of undiagnosed or misdiagnosed cases. Disease epidemiology is distorted; in fact, it can lead to the development of misinformed health policies with negative clinical and financial consequences [7,10].

The intersection of infectious disease mortality and gaps in primary healthcare and diagnostic capacity is a major concern. A premortem diagnostic pathway failure, particularly for diseases that present subtly, is underscored by the high rate of fatal infections found at postmortem. This is further complicated in rural areas and poorly resourced urban settings, where advanced diagnostics such as molecular tests or imaging analysis cannot be accessed [4,8]. Findings from autopsies, therefore, provide crucial audits on healthcare systems-not only revealing which pathogens are lethal but also where clinical detection fails; these should direct targeted investments into the most vulnerable parts of the health system, for example, point-of-care diagnostics for tuberculosis or sepsis biomarkers. Such insights ensure that policy interventions are data-driven and focused on closing specific gaps in the care cascade [10].

In South Africa, infectious diseases are a leading cause of death, but contemporary autopsy data on the spectrum of fatal infections in a tertiary care setting are scarce [11]. Thus, this study sought to conduct an autopsy-based study to define the full range of fatal infectious diseases in our context. This study aims to address this gap by presenting pathology-confirmed infectious disease cause-of-death data from a 10-year autopsy series at a single South African academic laboratory to inform local diagnostic protocols and mortality surveillance. The objectives were first, to provide data to support policies addressing these diseases at both hospital and community levels; second, to ensure accurate mortality data are channelled into national disease surveillance systems. In the end, findings should improve clinical vigilance by specifying patterns of infectious diseases that inform diagnostic protocols to achieve better patient outcomes.

## 2. Materials and Methods

This was a descriptive cross-sectional study based on a chart review, which involved a convenience sampling of all autopsies with the final cause of death as “infectious disease” from 1 January 2012 to 31 December 2021.

Cases were identified from the NHLS laboratory information system using a combined strategy. First, structured retrieval was performed using relevant SNOMED morphology (M) and topography (T) codes, selected previously based on the diagnostic entities of interest. These codes were used to extract histopathology records within the defined study period. Second, to minimise missed cases due to coding variability or incomplete coding, a supplementary free-text search of pathology reports was conducted using predefined keywords and synonyms such as “infectious diseases”, “bacterial disease”, “fungal disease”, “viral disease” and “parasitic disease”, corresponding to the study condition. Search words such as “mycobacteria”, “tuberculosis”, “TB”, “pneumonia”, “broncopneumonia”, “sepsis”, “meningitis”, “cryptococcus”, “cryptococcal”, “CMV”, “cytomegalovirus”, “HSV”, “herpes simplex”, “PCP”, “pneumocystis”, “candida”, “mucormycosis”, and “aspergillus” were also used as per the common diagnostic infection in our setting. The results from both approaches were merged, and duplicate records were removed.

All diagnoses included in the dataset were based on original histopathology reports issued by qualified pathologists in routine clinical practice. Within standard departmental diagnostic workflows, cases may undergo intradepartmental review, including peer discussion or second review by another pathologist in diagnostically challenging or complex cases, as part of routine quality assurance processes.

**Figure 1:**
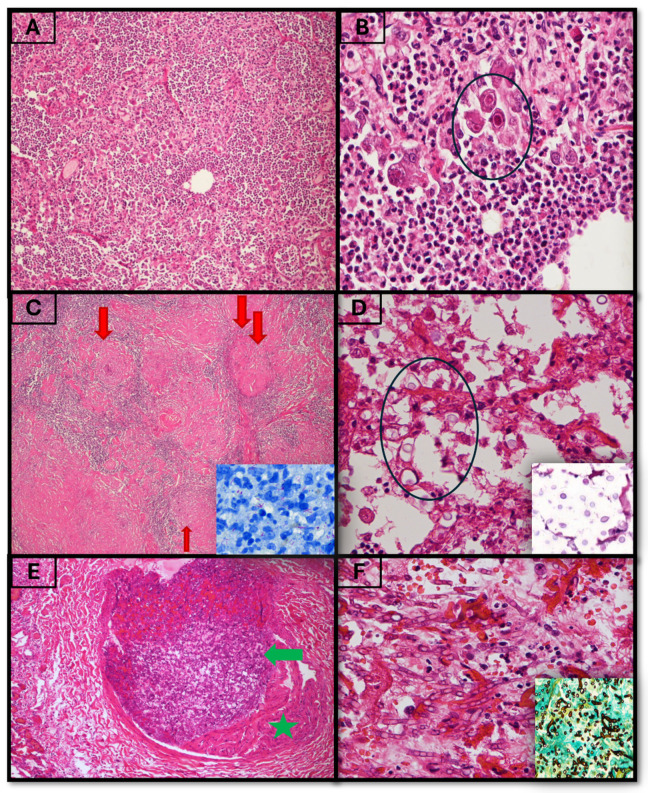
Microscopy of infectious diseases (H&E and special stains). (**A**,**B**) Cytomegalovirus in the lung (10× magnification). The circle shows cytoplasmic and nuclear inclusions of CMV, imparting an “owl’s eye” appearance (20× magnification). (**C**) Necrotising granuloma (red arrows) in the lung (4× magnification). Inset shows ZN stain with acid-fast bacilli. (**D**) Cryptococcal fungal yeasts (circle) (10× magnification). Inset shows PAS stain highlighting the fungi. (**E**,**F**) Mucormyocosis. (**E**) Fungal hyphae within a blood vessel (green arrow) in a lung (10× magnification). Green star shows vascular wall. (**F**) Higher magnification of fungal hyphae (20× magnification). Inset shows Grocott stain, which highlights the fungi.

The clinicopathologic characteristics, such as age, sex, comorbidities, HIV status, including CD4 count, viral load and treatment, clinical presentation, antemortem diagnosis and treatment information, and postmortem diagnosis, were retrieved from the histopathology reports. For the final postmortem diagnosis, gross descriptions of the organs were reviewed, using archived photographs when available, in conjunction with stored haematoxylin and eosin (H&E)-stained sections and histochemical slides. When H&E and histochemical slides were not retrieved, instead, tissues were re-cut according to the departmental standard operating procedure. Histochemical stains performed to highlight the microorganisms included the Ziehl–Neelsen (ZN) stain for mycobacteria, Periodic Acid–Schiff (PAS) stain for fungal organisms, Grocott’s Methenamine Silver (GMS) stain for fungal cell walls, Gram stain for bacterial organisms, and Mucicarmine stain for Cryptococcus neoformans capsules (Figure 1). The final cause of death was assigned through integration of antemortem clinical findings, and gross and histopathological autopsy findings. The inclusion criteria in this study were all clinical hospital autopsies of all age groups that were requested and performed within the study period who died from infectious diseases. Cases with a completed macroscopic (preliminary) autopsy report and cases with completed histological examination of sampled tissues were also included. All medicolegal and forensic autopsies and cases in which both the tissue blocks and slides or tissue block only were unavailable in the event where a case need histopathological reappraisal were excluded from the study. Due to the retrospective nature of this study, microbiological culture and sensitivity testing could not be done as they were not routinely requested at the time of perfoming the autopsy.

The collected data and results were entered electronically into Microsoft Excel (Microsoft Office 2016). A unique identifier was assigned to each case to prevent duplicate variables and maintain confidentiality. Double data capture was performed to avoid errors and ensure data reliability. The dataset was analysed using STATA-18 (Stata Corp., College Station, TX, USA), and a descriptive analysis was performed to calculate the prevalence of infectious disease.

## 3. Results

A total of 303 autopsies were performed during the 10-year study period and were screened for eligibility. Of these, cases were sequentially assessed for inclusion based on predefined criteria.

Following the screening process, cases were excluded if they did not meet the inclusion criteria. After applying the inclusion and exclusion criteria, 55 cases met all eligibility requirements and comprised the final study cohort.

The final analytical sample, therefore, consisted of 55 autopsy-confirmed infectious disease deaths drawn from an initial pool of 303 autopsies performed during the study period.

The study cohort consisted of 37 females (67%) and 18 males (32%). The median age was 31 years, with an interquartile range (IQR) of 19–45 years. There were 10 (18%) paediatric decedents (under the age of 14). The minimum age was two months, and the maximum age was 74 years.

### 3.1. Infectious Disease

**Figure 2:**
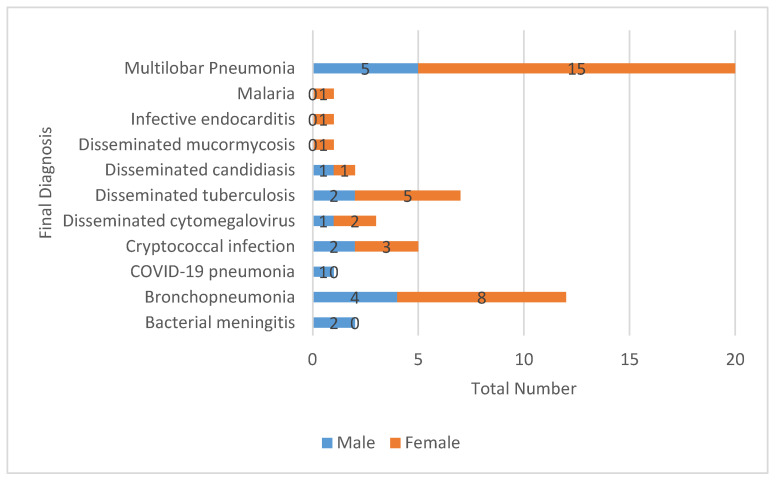
Illustration of the final diagnosis characteristics with a gender split.

The distribution of infectious diseases revealed that 19/55 (35%) of decedents were HIV-positive, 33/55 (n = 60%) were HIV-negative, and the status was unknown for 3/55 (6%). Among HIV-positive individuals, 15/19 (79%) were female, and 13/19 (68%) were receiving antiretroviral therapy (ART) (Figure 2). The median age of the HIV-positive subgroup was 31 years (IQR 23–37), with one paediatric case of a 7-month-old. The leading autopsy-confirmed cause of death was multilobar pneumonia 20/55 (37%), followed by bronchopneumonia 12/55 (22%).

### 3.2. AIDS-Defining Illness

AIDS-defining illnesses were present in 15/55 (27%) of cases. Tuberculosis was the most common, accounting for nearly half of these cases, 7/15 (46%), followed by cryptococcal infection, 5/15 (33%). Other disseminated opportunistic infections included cytomegalovirus, 3/15 (20%). Among patients with AIDS-defining illnesses, 11/15 (73%) were female.

### 3.3. Septic Shock

Septic shock was identified in 10/55 (18%) of decedents, 8/10 (80%) of whom were female. While the majority were adults, one case was a five-year-old child. Underlying fatal infections in this subgroup included multilobar pneumonia, tuberculosis, bacterial meningitis, disseminated candidiasis, disseminated cytomegalovirus, disseminated mucormycosis, and cryptococcal infection, as shown in Table 1 and Figure 1.

### 3.4. Final Diagnosis Characteristics with Gender Split

Multilobar pneumonia was the most common diagnosis, affecting 20/55 (36%) of participants, with 15/55 (27%) females and 5/55 (9%) males. Bronchopneumonia was the second most frequent diagnosis, observed in 12/55 (22%) of cases, with females predominating (8/55, 15%) compared to males (4/55, 7%). Tuberculosis occurred in 7/55 (13%) and cryptococcal infection in 5/55 (9%). Overall, females had higher frequencies across most diagnoses. Figure 2 provides further reference.

## 4. Discussion

This autopsy-based study provides a critical, pathology-confirmed window into the causes of infectious mortality in a contemporary South African setting. The findings in this study reveal a complex and continuing interplay of HIV, tuberculosis, and other opportunistic infections. These findings underscore the unfinished and evolving nature of the HIV epidemic and highlight the gradual challenges within a transitioning healthcare terrain.

This study showed female predominance (67%) with a young median age (31 years), which is typical of the impact of the HIV epidemic in sub-Saharan Africa. This aligns with both the national and African regional data that show HIV-related mortality predominantly affects women during their reproductive age. This imbalance could be linked to biological, social, and structural vulnerabilities [12,13].

The finding that 35% of all decedents were HIV-positive establishes HIV as a fundamental underlying determinant of mortality in this population. More critically, the fact that 68% of these HIV-positive individuals were documented to be on ART is a significant and concerning observation. It shows a move from the pre-ART era, where lack of treatment contributed to the majority of deaths, in contrast to the current era, where most people with HIV are on treatment, including those with advanced HIV disease [14]. This study suggests that a considerable section of fatalities due to HIV now happen in people who may have accessed the healthcare system; however, they may have initiated treatment late or have failed therapy to prevent fatal immunosuppression and associated complications. In this cohort, these findings point to potential priorities for local health policy including bolstering point-of-care diagnostics for sepsis and TB, increasing cryptococcal antigen screening at CD4 thresholds, and including autopsy findings in hospital mortality review committees as a quality improvement tool [15,16].

This assertion is evidenced by the high burden of AIDS-defining illness, which was seen in 27% of all cases, which comprised the majority of deaths in HIV-infected individuals. The spectrum of AIDS-defining illness reflects the known burden of opportunistic pathogens in South Africa [17].

Tuberculosis is attributed to half of the AIDS-defining illness cases, confirming its position as the leading cause of death among people living with HIV (PLWH) in South Africa and globally [12]. The continuation of TB and disseminated disease highlights TB prevention gaps, diagnosis, and management of drug-resistant strains.

The second most common AIDS-defining illness is cryptococcal infection, which is a prototype of advanced immunosuppressed disease and constitutes approximately 20% of global HIV-related deaths, especially cryptococcal meningitis [18]. The presence of cryptococcal infection in this study underscores the critical need for routine cryptococcal antigen screening, especially in individuals with low CD4 counts. Cryptococcal serum antigen is a cost-effective intervention recommended for individuals with a CD4 count ≥ 100 [19].

Although AIDS-defining illness were prominent, the leading immediate causes of death in this study were multilobar pneumonia (36%) and bronchopneumonia (22%). This predominance of lower respiratory infections is consistent with global burden of disease data, which identify lower respiratory tract infections as a leading cause of mortality [17]. Although pathological tissue reaction microscopically supports multilobar pneumonia and bronchopneumonia, in the absence of routine antemortem microbiological confirmation, the bacterial aetiology of pneumonia in this cohort remains hypothetical [20,21]. They may be community-acquired bacterial pneumoniae secondary to *Streptococcus pneumoniae* and *Haemophilus influenzae*, amongst others, which have a higher morbidity and mortality rate in people living with HIV, although they can be seen in HIV-negative individuals. The high prevalence of these pneumonias may highlight broader systemic factors, such as poverty, including overcrowding and air pollution, which predispose these individuals to severe respiratory infection [22,23].

The severity of these infections is clinically shown by the prevalence of septic shock at 18%, which is a terminal event. The aetiologies of shock in this study were diverse, including bacterial pneumonias and meningitis, disseminated fungal infections (candidiasis, mucormycosis), and viral infections (CMV, HSV). These findings may be due to the state of deep immune dysfunction in these individuals who are prone to disseminated and severe diseases [24]. The predominance of females among septic shock decedents is notable and requires further investigation. The presence of a paediatric case (5 years old) in septic shock underscores the susceptibility of children to severe infections.

Comorbidities observed in this cohort are consistent with local observations and correspond to the broader documented epidemiologic transition in South Africa, where HIV, TB and non-communicable diseases are increasingly co-occurring [25,26]. While 49% of decedents had no recorded major comorbidity, a significant proportion suffered from non-communicable diseases (NCDs): hypertension (18%), diabetes mellitus (8%), and malignancy (11%). This reflects the “double” or even “triple” burden of disease facing South Africa, where HIV, tuberculosis, and NCDs like diabetes and hypertension increasingly coexist [27]. This syndemic, where these diseases cluster and interact within a context of social and economic disadvantage, exacerbates outcomes. For instance, diabetes is a known risk factor for severe tuberculosis and poor treatment outcomes; similarly, HIV and some NCDs share inflammatory pathways that can accelerate end-organ damage [28,29,30]. The management of these intersecting conditions requires an integrated, patient-centred model of care that moves beyond vertical disease programmes, a transition that healthcare systems are still struggling to achieve [31].

The presence of paediatric decedents is a serious reminder of continued vulnerabilities amongst this age group. The seroconversion of a 7-month-old infant in this study suggests a failure and/or loophole in the prevention of mother-to-child transmission programme. This could be due to late antenatal booking by the mother, which may have led to a missed diagnosis, or to deficient compliance with antenatal ART. Additionally, there could have been challenges in postnatal prophylaxis and infant diagnosis. Generally, severe infections in the HIV-negative paediatric population point to deficient routine immunisation, accessible primary health care, and prompt treatment of diseases in children. Any paediatric death is a critical event which warrants a thorough social and healthcare systems analysis to mitigate future events [32].

The analytical power of our study is denied by several important limitations. The retrospective, single-institution study is subject to selection bias. The case cohort in this study represents individuals who died and underwent clinical autopsy, which may over-represent unexplained medical deaths. The sample size is small, which limits statistical power. The reported proportions reflect the distribution of infectious causes among autopsied deaths rather than population-level prevalence. As such, these findings should be interpreted within the context of a selected autopsy cohort, which may be subject to referral and selection biases, and may not be representative of the general population. Furthermore, the retrospective data collection was limited to incomplete information, as evidenced by missing HIV status in some cases, including the lack of ART information such as duration, adherence, viral load, and CD4 count. Although clinical autopsy may be used for definitive pathological diagnosis, it may not always identify the causative microorganism without ancillary microbiological studies, including molecular testing.

## 5. Conclusions

This autopsy pathology study elucidates the persistent burden of HIV and its complicating infections, especially TB and cryptococcosis, in a South African population. Some HIV-positive decedents were found to be on ART at the time of death, which leads to hypotheses of late ART initiation, poor adherence to treatment, or advanced disease at presentation. However, the absence of ART duration, adherence data, CD4 trends, and viral load information in this retrospective dataset precludes causal inference. The finding needs to be confirmed in prospective studies with complete clinical data.

These findings point to potential areas of focus for local public health strategy, including enhanced case finding and management of advanced HIV disease, pending confirmation in larger studies within the constraints of this small single-centre cohort.

Development of clinical protocols/guidelines and continuous professional development of healthcare workers should be undertaken to manage the conflux of HIV-related and non-related infections.

Future prospective studies with a stronger clinical-pathological focus, including appropriate ancillary studies, should help elucidate the specific pitfalls in the patient care cascade, with the ultimate goal of translating autopsy findings into actionable strategies for disease prevention and reducing patient deaths.

## Figures and Tables

**Table 1:** Demographic and clinicopathological data.

Variables	Frequency	Percentage (%)
Age		
Below 1 year	7	13
1–17 years	5	9
18–74 years	43	78
Race		
African	53	96
White	2	4
HIV Status		
HIV-Negative	33	60
HIV status Unknown	3	6
HIV-Positive	19	35
ART Status (among HIV-positive, n = 19)		
ART No	6	32
ART Yes	13	68
Comorbidities		
Congenital heart disease	1	2
Asthma	1	2
Diabetes Mellitus	5	8
Epileptic	1	2
Herbal intoxication and pregnancy	1	2
Hypertension	10	18
Malignancy	6	11
Myasthenia Gravis	1	2
Nephrotic syndrome	1	2
Previous Brain Surgery	1	2
Renal dialysis	1	2
Rheumatoid arthritis	1	2
Substance use (illicit drugs)	1	2
Thrombocytopenic purpura	1	2
None	27	49
Final Autopsy Diagnosis		
Bacterial meningitis	2	4
Bronchopneumonia	12	22
COVID-19 pneumonia	1	2
Cryptococcal infection	5	9
Disseminated cytomegalovirus infection	2	4
Disseminated tuberculosis	7	13
Disseminated candidiasis	2	4
Disseminated cytomegalovirus and HSV infection	1	2
Disseminated mucormycosis	1	2
Infective endocarditis	1	2
Malaria	1	2
Multilobar Pneumonia	20	36

Note: Percentages may exceed 100% as multiple comorbidities may be present in a single individual.

## Data Availability

The data that support the findings of this study are available from the corresponding author upon reasonable request.
